# Mitochondrial dysfunction and biogenesis: do ICU patients die from mitochondrial failure?

**DOI:** 10.1186/2110-5820-1-41

**Published:** 2011-09-26

**Authors:** Andrey V Kozlov, Soheyl Bahrami, Enrico Calzia, Peter Dungel, Lars Gille, Andrey V Kuznetsov, Jakob Troppmair

**Affiliations:** 1 Ludwig Boltzmann Institute for Experimental and Clinical Traumatology, AUVA Research Center, A-1200 Vienna, Austria; 2 Sektion Anästhesiologische Pathophysiologie und Verfahrensentwicklung, Universitätsklinikum, D-89070 Ulm, Germany; 3 Institute of Pharmacology and Toxicology, Department for Biomedical Sciences, Veterinary University Vienna, A-1210 Vienna, Austria; 4 Cardiac Surgery Research Laboratory, Department of Heart Surgery, Innsbruck Medical University, A-6020 Innsbruck, Austria; 5 Daniel Swarovski Research Laboratory, Department of Visceral-, Transplant- and Thoracic Surgery, Innsbruck Medical University, A-6020 Innsbruck, Austria

## Abstract

Mitochondrial functions include production of energy, activation of programmed cell death, and a number of cell specific tasks, e.g., cell signaling, control of Ca^2+ ^metabolism, and synthesis of a number of important biomolecules. As proper mitochondrial function is critical for normal performance and survival of cells, mitochondrial dysfunction often leads to pathological conditions resulting in various human diseases. Recently mitochondrial dysfunction has been linked to multiple organ failure (MOF) often leading to the death of critical care patients. However, there are two main reasons why this insight did not generate an adequate resonance in clinical settings. First, most data regarding mitochondrial dysfunction in organs susceptible to failure in critical care diseases (liver, kidney, heart, lung, intestine, brain) were collected using animal models. Second, there is no clear therapeutic strategy how acquired mitochondrial dysfunction can be improved. Only the benefit of such therapies will confirm the critical role of mitochondrial dysfunction in clinical settings. Here we summarized data on mitochondrial dysfunction obtained in diverse experimental systems, which are related to conditions seen in intensive care unit (ICU) patients. Particular attention is given to mechanisms that cause cell death and organ dysfunction and to prospective therapeutic strategies, directed to recover mitochondrial function. Collectively the data discussed in this review suggest that appropriate diagnosis and specific treatment of mitochondrial dysfunction in ICU patients may significantly improve the clinical outcome.

## ICU-related diseases

Patients admitted to the intensive care unit (ICU) are of different clinical etiology and characteristics. Many unplanned ICU admissions are for the treatment of cardiovascular disorders, which often are due to intraoperative complications, acute myocardial infarction, and coronary artery disease [1,2]. Trauma patients surviving massive bleeding constitute an additional ICU population with high risk of developing multiple organ failure (MOF) and mortality. Cardiovascular disorders, massive bleeding, and acute lung injury cause hypoxemia and tissue hypoxia. Hypoxia per se is thought to be a key factor in ischemic injury. Although ischemia associated with reduced oxygen supply is a life-threatening event and reperfusion with oxygenated blood is essential to interrupt hypoxia-induced cell death, the damaging effect of ischemia is not fully evident until reoxygenation [3]. Reperfusion injury implies some reactions initiated by reoxygenation of ischemic tissue [4,5]. This concept is supported by the observations that (a) little mucosal injury is detected during ischemia, but major changes occur after reperfusion [6,7] and (b) hypoxic reperfusion of ischemic tissue results in little additional damage [8]. In general, hypoxia-induced cell death appears after hours of ischemia, whereas the reperfusion injury may occur within minutes after reoxygenation [9-14].

Major burn injury and sepsis account for more than 25% of all ICU admissions [15]. In general, the body's response to an initial insult, e.g., trauma, ischemia, burn, infection, or stress, is regulated by mediators derived from the activation of humoral cascades, such as complement, kallikrein/kinin, and coagulation systems and/or from a variety of cells, such as monocytes/macrophages, and the release of cytokines, proteases, oxygen radicals, and nitric oxide. Originally this response was developed to protect the host; however, beyond a certain threshold level of activation, it causes an imbalance of the mediator system that can harm the host by leading to the development of MOF. Although the pathogenesis of MOF is most likely multifaceted, some phenomena, such as ischemia/reperfusion associated with excessive free radical generation, activation and adherence of neutrophils to the endothelium and the subsequent transmigration into the surrounding tissue [16-20], gut barrier failure leading to the translocation of bacteria/endotoxin [21-28], and an initially hyperinflammatory state followed by delayed immune suppression that predispose to infection, have been considered key events in this scenario [29].

Despite early management and control of the acute phase by means of advanced ICU technology, a series of events may lead to failure of one or more organs (MOF) and finally death in some ICU patients. Acute lung injury (ALI) and acute respiratory distress syndrome (ARDS) remain a major problem. In 2005, the incidence of ALI and ARDS in adults was estimated to be approximately 200,000 patients annually in the United States, with a mortality of approximately 40% [30]. More recent reports on the ARDS-related mortality vary from 20-50% [31-36]. Acute traumatic coagulopathy (ATC) is observed in 10-25% of patients after major trauma and its management forms an integral part of hemostatic resuscitation [37]. Increasing severity of ICU patients associated with acute renal failure elevates mortality rate estimates by 15-60% [38-40].

It is commonly accepted that sepsis and ischemia-reperfusion (I/R) injury are among the leading causes of death in critically ill patients at the surgical intensive care unit setting [41]. Therefore, leading life-threatening pathological states in ICU patients are generally caused by impaired oxygen delivery followed by tissue ischemia or hypoxia and inflammation followed by excessive inflammatory response of the body. Depending on the location of these two processes, they cause dysfunction and failure of a corresponding organ, or fatal MOF, if they occur systemically. On the cellular level, there are two basic mechanisms causing organ dysfunction: cell death resulting in the reduction of cell numbers, and cellular dysfunction causing an accumulation of cells not able to support major organ functions.

Cell death is the main pathway of ischemia/reperfusion-mediated tissue damage and organ failure [42]. In contrast, a systemic inflammatory response causes organ dysfunction/failure often without remarkable cell death. Although inflammatory mediators have been shown to induce apoptosis and necrosis in some experimental models, the organs of animals or patients, even those who died of MOF, often appear normal [43] with neither major necrotic areas nor a relevantly increased number of apoptotic cells (except for lymphocytes) [44,45]. The most common change observed under both ischemic and inflammatory conditions is cellular stress accompanied by alterations in energy metabolism initiated at the mitochondria. This has been well documented in diverse experimental models. There is a body of data confirming that mitochondrial dysfunction occurs not only in experimental models but also in ICU patients. Mitochondrial dysfunction was determined in muscle biopsies from septic patients [46,47] and in peripheral blood monocytes [48]. Vanhorebeek et al. reported structural and functional abnormalities in liver but not muscle mitochondria from patients who had died in surgical critical care unit [49]. These findings are in line with data obtained in primates [50]. Mitochondrial dysfunction also was detected in liver biopsies of patients after liver transplantation [51], but it is not clear whether these changes are due to ischemia or inflammatory/immune responses of the host. In animal experiments, it has been shown that cold and warm ischemia accompanying heart and liver transplantation impair mitochondrial function [52-54].

## Mitochondrial dysfunction under hypoxia and inflammation

Impairment of oxygen delivery, inflammation, sepsis and other ICU-associated pathologies, all impose cellular stress and thus will affect profoundly mitochondrial physiology. Mitochondria have a variety of functions, which are not completely elucidated yet. Besides ATP synthesis, the best-known function of mitochondria, they are involved in several biosynthetic and signaling pathways. In context of acute critical diseases, the most important mitochondrial activities are oxidative phosphorylation and reactive oxygen species (ROS)-related signaling processes. Inhibition of oxidative phosphorylation (OXPHOS) and ATP synthesis impairs ion homeostasis, most importantly Ca^2+ ^homeostasis, frequently resulting in excessive ROS production originating both from mitochondrial and nonmitochondrial sources [55]. In addition, mitochondrial morphology and dynamics are affected, leading to the fragmentation of the mitochondrial network [56-58].

Several lines of evidence suggest that maintaining mitochondrial homeostasis and integrity is directly linked to cellular protection under conditions of cellular stress. In particular, the production of ROS is seen as the driving force behind mitochondrial dysfunction, playing an important role in the development of cellular malfunction and organ failure induced by inflammatory mediators and hypoxia [4,59-61]. Basic OXPHOS activity of mitochondria is controlled mainly by substrate and ADP availability and by the specific composition of respiratory supercomplexes in mitochondria [62]. Additional levels of control include allosteric regulation, reversible phosphorylation, and other forms of posttranslational modification. It has been proposed that phosphorylation is of special importance for controlling mitochondrial function [63]. The best evidence for such a mode of regulation is present in the case of protein kinase A (PKA) [64], which affects the activity of several OXPHOS enzymes and thereby serves to modulate ATP generation and ROS production. Notably, also dysfunction of OXPHOS enzymes correlates with clinical deterioration in sepsis [65]. Other primarily cytoplasmic signaling proteins have been suggested to regulate mitochondrial ROS production, both positively and negatively [66-68]. 

Finally, a major question remains: how pathologic stimuli are communicated to the mitochondria, affecting their function and how these organelles in turn orchestrate a cellular response to stress conditions. P66SHC (Src Homology 2 domain containing transforming protein) may be one candidate, whose action directly leads to the production of ROS under conditions of various cellular stresses. In this process, the primarily cytoplasmic protein translocates to the mitochondria in a mechanism involving protein kinase C (PKC) and prolyl-isomerase1 (Pin 1) [69,70]. Gene ablation experiments have highlighted the benefit of abrogating p66SHC in different pathological settings, such as aging or ischemia reperfusion injury [71]. Mitochondria also extensively communicate with the nucleus to assure proper cellular responses. Expression of nuclearly encoded genes is critical for mitochondrial protein synthesis and mitochondrial biogenesis by mechanisms, which include peroxisome proliferator-activated receptor-γ coactivator-1α (PGC-1α), nuclear respiratory factors (NRFs), and mitochondrial transcription factor A (mtTFA). Notably, ATP depletion activates mitochondrial biogenesis via AMP-activated protein kinase (AMPK) [72]. Factors released from mitochondria may constitute important signaling molecules in these processes. They also may include second messengers, such as ROS or Ca^2+ ^and the activation of signaling pathways downstream of mitochondria, which has been demonstrated in lower model organisms in the mode of retrograde signaling [73].

Very recently, it has been demonstrated that prevention of ROS production by mitochondria decreases inflammatory cytokines after cells stimulation by lipopolysaccharide (LPS), suggesting that mitochondrial ROS may be a therapeutic target for various inflammatory diseases [60]. Interaction of cytokines with their target cells involves cytokine receptors, which activate intracellular signaling cascades. Universal and essential to cytokine receptor signalling is the JAK-STAT (JAK = Janus-Kinase, STAT = Signal Transducers and Activators of Transcription) pathway. Almost 40 cytokine receptors signal through combinations of four JAK and seven STAT family members, suggesting commonality across the JAK-STAT signaling system [74-76]. Some also activate NF-kB, stress-kinase pathways, or the Ras-ERK (Ras = Rat sarcoma; ERK = extracellular signal-regulated kinases) pathway [77,78].

In addition, it has become clear that mitochondria can be considered as an important source of damage-associated molecular patterns (DAMPs) for the activation of innate immunity. Mitochondrial proteins and DNA released from damaged necrotic or apoptotic cells may activate mitochondrial DAMPs-mediated inflammation (sterile inflammation) during ischemia-reperfusion of various organs. Similarly, mitochondrial DAMPs can be released in patients with infections, contributing thus to the pathological mechanisms of sepsis [65,79-81].

Therefore, mitochondria seem to be the key players in disorders induced by ischemia and inflammation in ICU patients. The pathologic impact of mitochondria results from the depletion of ATP, release of proapoptotic proteins, excessive production of ROS, and disturbance in Ca^2+ ^homeostasis. Below, we consider how these events will be manifested on the cell/organ level.

## Pathological consequences of mitochondrial dysfunction

### ATP depletion

Mitochondrial ATP synthesis is regulated by substrate supply and by the coupling of phosphorylation to the proton gradient generated by mitochondrial electron transfer and by the demand in ATP. Coupling of phosphorylation to the proton gradient is usually regulated by uncoupling proteins (UCP) as well as physicochemical variations of the inner mitochondrial membrane and can be disrupted by the opening of the mitochondrial permeability transition pore under pathological conditions. Although increased mRNA levels for UCPs in mouse liver were detected during sepsis, so far there is no unequivocal evidence that they are linked to mitochondrial uncoupling under such conditions [82]. This fits to the observation that respiratory control values of rat liver mitochondria were identical or even better than those of control animals [83]. ATP depletion accompanied with an inhibited Na/K pump leads to an increase in cellular Na concentration, which results in cellular gain of electrolytes and water, causing early reversible cell swelling [6,84]. A prolonged period of hypoxia is then followed by a loss of mitochondrial matrix and disintegration, expansion and formation of vesicles in the endoplasmatic reticulum and cytoplasm, and lysosomal rupture with release of enzymes as a final step of cell death [6,85]. Besides direct damage of mitochondria, the reduced supply with NAD+(H) as substrate, which is consumed by an increased poly(ADP-ribose)polymerase (PARP) activity for DNA repair under conditions of sepsis was suggested [86]. However, there was no recent development regarding the impact of PARP in ICU-related diseases. For direct mitochondrial damage, ROS and RNS have been favored [87]. Eventually, however, continuing adverse stress stimuli will result in cell death through apoptosis or necrosis, the latter is commonly a result of insufficient ATP provision [88,89]. Apart from cell death, an insufficient production of ATP may result in cellular dysfunction. It has been shown that in animal models of severe inflammation the ATP levels were halved compared with controls [90]. Similar results were reported in septic patients; approximately halved ATP levels were found in the "non-survivors" group compared to "survivors" group [47].

### Cytochrome c and AIF release

Irrespectively of the trigger, impairment of mitochondrial function, often associated with a drop of the mitochondrial membrane potential, is followed by a release of proapoptotic factors, such as cytochrome c from the intermembrane space with subsequent activation of caspases [91,92]. Although the involvement of the mitochondrial permeability transition pore in this event was discussed, the precise mechanism is still unclear. Inflammation-triggered lipid peroxidation also was related to mitochondrial dysfunction. It has been argued that ROS-induced cardiolipin oxidation decreases its close association with cytochrome c and causes a higher mobility of cytochrome c facilitating its release from mitochondria [93], probably via the upregulation of Bax, a proapoptotic protein, building the channels in outer mitochondrial membrane. Upregulation of Bax in yeast was associated with an increased amount of oxidized lipids [94]. This, however, was not confirmed in mammalian cells yet. Another protein that induces apoptosis is apoptosis-inducing factor (AIF). Proapoptotic activity of AIF is associated with the increase of intracellular Ca^2+ ^(e.g., ischemia/reperfusion injury). Increased intracellular Ca^2+ ^levels in turn trigger the depolarization of the mitochondrial membrane with subsequent loss of membrane potential and elevated generation of ROS [95,96]. AIF-mediated induction of apoptosis requires its further translocation to the nucleus to induce DNA degradation [97].

### Reactive oxygen and nitrogen species, carbon monoxide

ROS are a group of molecules with widely differing reactivity and damaging potential in biological systems. The technical inability to differentiate easily individual ROS by most analytical methods resulted in many contradicting results in this field. Interpretation of ROS effects should always take into account that different ROS may have completely different biological effects. Certain ROS (such as HO· and ROO·) themselves can directly damage biomolecules at high levels (e.g., cardiolipin oxidation), whereas the same ROS and also other ROS species at lower concentrations modulate protein function through redox-modification [79]. Thus, limiting ROS production or lowering ROS levels through the use of antioxidants seems to be a straight forward approach to reduce damage induced by ischema/reperfusion or inflammation. However, in the clinical setting their use had little benefit in limiting ROS-associated tissue and organ damage. Due to diverse chemical properties of individual ROS and the various sites of their formation, their detoxification can be limited by the availability of appropriate antioxidants at these locations. Applications of diverse antioxidants and ROS scavengers will be discussed below.

An alternative strategy, whose feasibility is supported by increasing experimental evidence, is to modulate mitochondrial ROS production itself. This may be achieved through activation or inhibition of intracellular signaling pathways, which have been implicated in the regulation of mitochondrial ROS production but also other proteins can be of interest for therapeutic intervention. These include mitochondrial uncoupling proteins (UCPs), which reside in the inner mitochondrial membrane and govern mitochondrial membrane potential (ΔΨ_m_) and, therefore, ROS generation and Ca^2+ ^influx [98]. Also, the prevention of mitochondrial fragmentation in cardiomyocytes protected hearts against ischemia/reperfusion injury [99]. Additionally, intracellular signaling pathways may modulate ROS production as discussed above, opening the possibility for future therapeutic interventions.

Nitric oxide (NO) formed under inflammatory and hypoxic conditions is an important modulator of mitochondrial function. We discuss this issue very briefly, because this topic has been reviewed extensively in the past [100-102]. It is commonly accepted that under normoxic conditions NO is synthesized by constitutive and inducible forms of NOS (cNOS and iNOS, respectively). Upon inflammatory response, iNOS is upregulated by specific proinflammatory agents, such as endotoxin, tumor necrosis factor alpha (TNF-alpha), interferon-gamma (IFN), and interleukin-1 (IL-1) in certain cells resulting in an excessive NO production (reviewed in [100,103-105]). In contrast to inflammation during ischemic/hypoxic conditions, NOSs are less efficient because their enzymatic activity requires oxygen and NO is generated via oxygen independent reduction of nitrite (reviewed in [102]). The mitochondria are one of the major targets for NO. NO itself reversibly inhibits the mitochondrial respiratory chain at complex IV, whereas peroxynitrite formed from NO and superoxide radical inhibits mitochondrial respiration at multiple sites and also causes mitochondrial permeability transition (reviewed in [101,106]).

Carbon monoxide (CO) is another gas messenger that controls mitochondrial function. CO has been shown to stimulate mitochondrial biogenesis; there is evidence that CO signalling is mediated by mitochondrial ROS (reviewed in [107]). CO also has been shown to modulate immune response stimulating production of anti-inflammatory cytokines [108]. Together, these data suggest that NO/CO may be beneficial or deleterious and only controlled low amounts of these gas messengers exert beneficial effects.

## Biogenesis of mitochondria and autophagy

The data on mitochondrial dysfunction under ischemic and inflammatory conditions are sometimes contradictory. Thus, upon diverse pathologic conditions involving systemic immune response different groups reported impaired [50,109-111], unchanged [112-114], and even improved [115-117] mitochondrial function. This variation may relate to experimental conditions, such as the severity of the insult, the duration of the study, and others. Another possible explanation for these conflicting findings may be the activation of natural adaptive reactions designed to restore mitochondrial function. They include biogenesis of mitochondria [118] and autophagy, which removes damaged mitochondria [119].

The importance of mitochondrial biogenesis after hypoxia has been shown in a variety of organs. Ahuja et al. [120] demonstrated that in the heart pathological stressors, such as ischemia, are associated with the downregulation of mitochondrial biogenesis via PGC-1 activity. Also the transcription factor Myc may play a key role in regulating cardiac metabolism and mitochondrial biogenesis in response to pathological stress. Myc activation in the myocardium of adult mice increases glucose uptake and utilization, downregulates fatty acid oxidation by reducing PGC-1alpha levels, and nevertheless induces mitochondrial biogenesis [120]. In the liver Wyatt et al. [121] studied the role of hexokinase III (HKIII), an important enzyme in glucose metabolism. Nuclear factor (erythoid-derived2)-like2, also known as NFE2L2 or Nrf2, which is involved in increasing the levels of endogenous antioxidants and attenuating apoptosis, has been shown to induce mitochondrial biogenesis [122]. HKIII is regulated by hypoxia and exerts protective effects against oxidative stress, perhaps by increasing ATP levels, reducing oxidant-induced ROS production, preserving mitochondrial membrane potential, and increasing mitochondrial biogenesis. In the kidney several studies showed that PGC-1alpha is an important regulator of mitochondrial biogenesis. In a model of oxidative injury mimicking ischemia-reperfusion damage, Rasbach et al. [123] showed that increased mitochondrial biogenesis accelerated recovery of mitochondrial function, mediated by p38 and epidermal growth factor receptor (EGFR) activation of PGC-1alpha. In another study, they demonstrated that mitochondrial biogenesis is mediated via 5-HT receptors and suggest that 5-HT-agonists may be effective for the treatment of mitochondrial and cell injury [124].

Mitochondrial biogenesis in sepsis is stimulated by the elevated production of NO and ROS, which leads to oxidative damage of mitochondrial DNA (mtDNA) and initiates a complex crosstalk between mitochondria and nucleus promoting an increased synthesis of new organelles [125]. Suliman et al. for the first time demonstrated that lipopolysaccharide stimulates mitochondrial biogenesis in rat hearts in response to oxidative cell damage [126,127]. In another study, they also showed that this simultaneous occurrence of mtDNA damage and compensatory mitochondrial biogenesis under heat-inactivated *E. coli *exposure results from the activation of toll-like receptor 4 (TLR-4) [128]. In a more recent study, the same group demonstrated that mitochondrial biogenesis is capable to restore oxidative metabolism in an experimental model of murine peritonitis, thus providing a potential mechanism affecting sepsis outcome [129]. Indeed, experimental and clinical data clearly suggest that mitochondrial dysfunction is closely linked to the onset of multiple organ failure in sepsis and the capacity to resolve this condition may depend on the ability to restore an adequate mitochondrial function [130]. In other terms, the failure of maintaining mitochondrial function through biogenesis may contribute to bad outcome. Indeed, a recent report provides first evidence that the activation of mitochondrial biogenesis may affect survival in critical illness [131]. Accordingly, the search for strategies to maintain and protect mitochondrial biogenesis has been proposed as an innovative research direction potentially providing new ways for preventing the onset of multiple organ failure in septic patients [125].

Damaged mitochondria are removed from cells by means of autophagy. Autophagy has been shown generally to limit cellular damage and cell death, appearing as a cell-survival response [132]. Autophagy is an evolutionary conserved process that involves a complex sequence of vesicle formation and fusion with lysosomes leading to the degradation of cellular structures and the recycling of end products [133]. It also has been shown that autophagy can be directly triggered by ROS [134,135].

## Possible therapeutic strategies to modulate mitochondrial function

### Antioxidants

Cellular and organ dysfunction related to the damage of lipid membranes and membrane-bound proteins by oxygen radicals is a rationale for the treatment of sepsis and septic shock by lipophilic antioxidants. The lipophilic antioxidants, which are most relevant in this context, are compounds of the vitamin E group (tocopherols and tocotrienols) and ubiquinones. The structure of such molecules usually consists of a redox-active part and a lipid anchor [136]. The redox-active part corresponds to the chromanol and benzoquinone head group for vitamin E compounds and ubiquinones, respectively. The lipid anchor is a C16 residue in vitamin E and an isoprenic side chain for ubiquinone. The most frequent types of these compound groups in mammalian tissues are alpha-tocopherol and ubiquinone-10 in humans. In rats, which are frequently used in septic shock models, ubiquinone-9 predominates. Although vitamin E compounds are antioxidants per se due to their phenolic OH group, ubiquinone needs to be reduced to ubiquinol (hydroquinone form) before it is active as an antioxidant. The benefit of vitamin E and ubiquinone-related antioxidants under conditions of ischemia/reperfusion was demonstrated in different experimental models [137-139].

Besides their function as antioxidants, the effects of vitamin E compounds on several signaling factors [140] and the function of ubiquinone as electron carriers in mitochondria are other important biological activities. Because ubiquinones are continuously synthesized in all tissues and both vitamin E and exogenous ubiquinone are continuously supplied by the diet, the primary question is whether there is an increased demand for such compounds during sepsis and septic shock. There have been several reports about the increase of ROS and lipid peroxidation under such conditions. However, only a few reports demonstrate clinically the decrease of lipophilic antioxidants in the plasma of septic patients. Some authors demonstrated that alpha-tocopherol levels in plasma are decreased in septic patients [141,142]. On the other hand, it was shown that among septic patients alpha-tocopherol levels did not differ between patients developing MOF and other patients [143]. Furthermore, in septic shock patients increased plasma levels of bilirubin were shown to counterbalance the loss of typical lipophilic plasma antioxidants, such as tocopherols and ubiquinones [144]. Little is known about concentrations of ubiquinone in the plasma of those patients. However, from the fact that sepsis was linked to increased glycolysis and mitochondrial dysfunction, the supplementation with ubiquinone to support mitochondrial functions seems to be logical and is supported by some clinical reports [145-148]. In addition, the suggested use of statins, which target the HMG-CoA reductase, against inflammatory cascades initiated during sepsis [149] provides another link to ubiquinone supplementation. It is well-known that the use of statins results in a decrease of cellular ubiquinone concentrations, which is possibly associated with adverse effects of statins [150]. Based on this relationship, ubiquinone supplementation under conditions of sepsis to prevent adverse effects of statins may be a reasonable idea to explore. However, currently there are no data on that. Whereas supplementation of ubiquinone and vitamin E in low concentrations is rather harmless, high concentrations and long-lasting application have been shown to increase the risk of bleeding in patients due to their anticoagulant effects [136,151].

### Mitochondria-targeted antioxidants

Because mitochondrial dysfunction has been shown to play a major role in hypoxia-mediated injury and mitochondria are the major cellular source of ROS, there is considerable interest in targeting antioxidants to mitochondria [152]. However, the benefit of this strategy is still debated because the results are not clear.

In a cellular model of I/R, Loor et al. [153] demonstrated that antioxidant administration during ischemia prevented the release of cytochrome c and calcium to the cytosol, which are known to contribute to I/R damage. However, in a study on neuronal survival in the rat striatum after acute perinatal hypoxia-ischemia, no significant difference was seen between MitoQ-treated animals and their respective vehicle-treated controls [154]. Lowes and co-authors showed that MitoQ may be beneficial in sepsis protecting mitochondria from damage and suppressing the production of the anti-inflammatory mediators [155]. Thus, this therapeutic strategy to prevent mitochondrial damage has to be further investigated. Mitochondrial targeting using peptide mimetics or lipophilic cationic agents (MitoQ, SkQ1) may offer improved antioxidant therapies. However, these compounds also have to be directed to the respective organ or cell type, which may be difficult [65].

### Donors of NO and CO

In the progression of I/R injury mitochondrial dysfunction, characterized by depletion of ATP, calcium-induced opening of the mitochondrial permeability transition pore, and exacerbated ROS formation play a key role [156]. Recently, nitrite was recognized as a nitric oxide (NO) donor specifically in hypoxic/acidic conditions, without substantially altering otherwise normal tissue that mediates cytoprotection after IR. The benefit of nitrite treatment has been shown in various *in vivo *models and organs [157-159]. Whereas most studies so far have investigated the effects of bolus treatments with nitrite, a recent paper by Jung et al. showed that long-term nitrite therapy, when initiated 24h after I/R, corrected the subacute hostile environment, induced tissue and vascular regeneration, and improved functional recovery [157,159,160]. Thus, they concluded that early and subsequent long-term nitrite therapy may be effective for the management of ischemic conditions, e.g., in stroke patients.

Endogenous NO is very diffusible and has many effects on mitochondrial physiology. Normal levels of intracellular NO stimulate mitochondrial biogenesis via cGMP and PGC-1 [161,162]. NO produced by eNOS activates cGMP generation from soluble guanylate cyclase. This leads to the expression of the transcriptional coactivator PGC-1, increasing production of NRF-1 and thus activating mitochondrial biogenesis [162]. Moreover, NO affects vascular smooth muscles, leading to vasodilation. This should be associated with improved availability of substrates and oxygen for cells and mitochondria. However, during inflammation, high levels of NO are produced due to activated expression of iNOS [163,164]. This elevated production of NO under pathological conditions directly inhibits mitochondrial respiration mostly through inhibition of respiratory complex IV (cytochrome c oxidase, COX) remarkably reducing OXPHOS [101,165]. This may lead to incomplete reduction of oxygen, increasing production of ROS and activating AMPK. Moreover, NO reacts with superoxide producing cytotoxic peroxynitrite damaging mitochondria [166,167]. It has been shown that under hypoxic conditions nitrite-derived NO, inhibits complex I, but ameliorates oxidative inactivation of complexes II-IV and aconitase during reoxygenation, thus preventing mitochondrial permeability transition pore opening and cytochrome c release [168]. Similar effects were found with a CO donor, tricarbonylchoro (glycinato)ruthenium, which act similar to NO targeting metalloproteins. Lancel at al. have shown in a sepsis model that CO stimulates mitochondrial biogenesis and reduces mortality in septic mice [169].

### Hydrogen sulfide (H_2_S)

During the past few years, H_2_S has been rediscovered as a physiological mediator potentially involved in several cellular processes [170,171]. A growing body of evidence seems to confirm the capacity of this molecule to protect organ functions from ischemia/reperfusion injuries [172,173]. In contrast, the role of sulfide in inflammation and sepsis is still a matter of debate. In fact, marked pro- [174,175] as well as anti-inflammatory effects of H_2_S [176-178] have been observed in different experimental studies. In principle, a link between H_2_S-exposure and mitochondrial function is given by the well-known capacity of sulfides to strongly inhibit the cytochrome c oxidase (COX) [179], i.e., the final electron acceptor of the mitochondrial respiratory chain. This property mainly determines the high toxicity of this compound. On the other hand, this capacity does not imply that the biological properties of H_2_S are exclusively mediated through direct effects on the mitochondria [170]. For example, H_2_S-therapy has been observed to preserve mitochondrial function in the heart muscle after I/R injury [176,180], but it failed to increase mitochondrial biogenesis [180]. Based on these observations, it was argued that H_2_S mainly prevents mitochondria from damage through an antioxidative effect. Accordingly, antioxidant effects of sulfide therapy have been demonstrated in the kidney after I/R injury [181]. Furthermore, interactions between H_2_S and potassium-dependent ATP-channels [182] as well as an eventual modulation of NO- and CO-related effects by H_2_S [183] are discussed as potential mechanisms of action at cellular level. Calvert et al. investigated the effect of exogenous hydrogen sulfide on survival rate in response to myocardial ischemia, which was induced by subjecting mice to permanent ligation of the left coronary artery for 4 weeks or to 60 minutes of left coronary artery occlusion followed by reperfusion for 4 weeks [180]. H_2_S therapy increased the phosphorylation of protein serine/threonine kinase B (PKB, AKT) and increased the nuclear localization of two transcription factors, nuclear respiratory factor (NRF) 1 and NRF2, which are involved in increasing the levels of endogenous antioxidants, attenuating apoptosis, and increasing mitochondrial biogenesis [180].

### Pyruvate

Pyruvate is an activator of the pyruvate dehydrogenase complex (PDHC) and reduces the cytoplasmic NADH/NAD+ ratio by stimulating the glycolytic pathway [184]. It has been proposed recently as a potential therapeutic in mitochondrial diseases and it was indeed effective in the treatment of a patient with Leigh syndrome due to cytochrome c oxidase deficiency [184,185]. Because an inhibition of the PDHC has been observed in sepsis, a similar benefit of pyruvate-therapy may be expected in this condition [186,187]. Indeed, indirect evidence for the potential effects of pyruvate in sepsis is provided by experiments conducted with dichloroacetate (DCA), a structural analogue of pyruvate that also activates the PDHC. As predicted by the mechanism of action of DCA, this treatment allowed reversing the disturbed lactate and glucose metabolism in septic animals [188]. However, a controlled, clinical trail of DCA for the treatment of patients with sepsis or liver failure did not show beneficial effects to improve hemodynamics or survival [189]. Additionally, pyruvate also acts as an antioxidant, and its pharmacologic potential in sepsis as well as in other critical conditions seems to be partially related to this property [190,191].

### Cytochrome c

Exogenous cytochrome c administration has been proposed as a further therapeutic approach for mitochondrial dysfunction in sepsis [192]. The rationale for this treatment is given by the observation that cardiac depression in septic animals developed simultaneously to the onset of COX-inhibition. Indeed, exogenous cytochrome c administration was shown not only to replete cardiac mitochondria with substrate and to increase COX-activity level but also improved cardiac function in septic mice [193]. In a further experimental study, these effects were observed up to 72 hours and even survival of the animals was improved [194]. However, no data are available to support the potential benefits of exogenous cytochrome c in humans.

### Preconditioning

Preconditioning is a phenomenon in which protection against severe injury is achieved by adapting to low doses of insults (reviewed in [195,196]). Preconditioning stimuli include ischemia/hypoxia, low doses of endotoxin, adenosine A1 agonists, opioid delta1 agonists and others. Sublethal ischemia, however, leads to cellular alterations, termed "hypoxic priming," of second messengers, such as cellular ionized calcium (Ca^2+^), cyclic cAMP, phosphatidic acids, and ROS [197-200]. Cells primed during ischemia are more susceptible to further release of ROS subsequent to reperfusion and active participants in the inflammatory response. Mitochondria are believed to be the end target for preconditioning operating via NO signaling pathways [201] or stimulation of mitochondrial biogenesis [202].

### Illumination and lasers

Low-level laser therapy (LLLT) has been found to biostimulate various biological processes, such as attenuation of ischemic injury. Avni et al. showed in a model of I/R injury in the gastrocnemius muscle in rats that LLLT significantly prevented degeneration after I/R, probably by induction of synthesis of antioxidants and other cytoprotective proteins [203]. A probable mechanism of light was shown by Dungel et al. who demonstrated that mitochondrial respiration inhibited by NO could be efficiently restored by illumination in a wavelength-dependent manner [204]. This effect was used by Mittermayr et al. who used blue laser irradiation of NO-Hb in the blood to cause decomposition of NO-Hb complexes and to release free NO. This led to a clear enhancement of local tissue perfusion decreasing the ischemic area in a skin flap model in rats [205].

### Side effects of ICU therapy on mitochondrial function

Antibiotics are one of the most common therapies administered in the intensive care unit setting. In addition to treating infections, use of antibiotics contributes to the emergence of resistance among pathogenic microorganisms (reviewed in [206]). Several classes of antibiotics function by binding to the bacterial ribosome and inhibiting bacterial protein synthesis. The mitochondrial protein synthesis machinery is in many ways similar to the prokaryotic machinery and as a result may be a target for antibiotics [207,208]. For instance, oxazolidinones that were very potent as antibiotics are uniformly potent in inhibiting mitochondrial protein synthesis [209]. This suggests considering antibiotics and other therapies used in ICU with respect to their impact on mitochondrial function.

## Conclusions

The exact mechanisms causing death of ICU patients are still not fully understood, although it is commonly accepted that single or multiple organ failure are the major reasons for death. Mitochondria play an important role in the development of malfunction of various organs, such as heart, liver, and kidney, in a mode that involves changes in turnovers of ATP, ROS, Ca^2+^, and release of proapoptotic proteins. Cell stress occurring under ischemia or/and inflammation always present in ICU patients compromise mitochondrial function, which contributes greatly to the metabolic changes, resulting in cell dysfunction and death, which in turn cause organ failure. Mitochondria can directly (e.g., via decrease in ATP levels) and/or indirectly (e.g., via modulation of ROS-dependent signaling) contribute to cellular dysfunction and death causing organ failure contributing to fatal outcome of ICU patients. Therefore, strategies to prevent mitochondrial injury in clinically relevant settings may provide new therapies for ICU-related disorders. Current therapeutic options include antioxidant therapy, nitric oxide donors, and low-level laser therapy, but further studies are necessary to clarify possible benefits for ICU patients. Targeted antioxidants and nitrite are in the phase of clinical trials (NCT00329056 and NCT00069654, respectively). Thus, there is a solid body of data that suggest significant contribution of mitochondrial dysfunction to outcomes of ICU patients. This, together with the fact that a number of relevant therapeutic tools have been developed within the last decade, suggests focusing more on detection and specific treatment of mitochondrial dysfunction in ICU patients to improve the clinical outcome.

## List of abbreviations

AIF: apoptosis-inducing factor; ALI: acute lung injury; AMPK: AMP-activated protein kinase; ARDS: acute respiratory distress syndrome; ATC: acute traumatic coagulopathy; DAMPs: damage-associated molecular patterns; ERK: extracellular signal-regulated kinases; HKIII: hexokinase III; 5-HT: 5-hydroxytryptamine receptors; I/R: ischemia-reperfusion; ICU: the intensive care units; JAK: Janus-Kinase; MOF: multiple organ failure; mtDNA: mitochondrial DNA; mtTFA: mitochondrial transcription factor A; Nrf2: factor-E2-related factor; NRFs: nuclear respiratory factors; OXPHOS: oxidative phosphorylation; P66SHC: Src, homology 2 domain containing transforming protein; PARP: poly(ADP-ribose)polymerase; PGC-1α: proliferator-activated receptor-γ coactivator-1α; Pin 1: prolyl-isomerase1; PKA: protein kinase A; PKC: protein kinase C; Ras: rat sarcoma; ROS: reactive oxygen species; STAT: signal transducers and activators of transcription; TLR-4: toll-like receptor 4; UCP: uncoupling protein.

## Competing interests

The authors declare that they have no competing interests.

## Authors' contributions

AVKo has made substantial contributions to conception, analysis and interpretation of publications in the field of mitochondrial dysfunction, drafting the manuscript, revising it critically for important intellectual content, has given final approval of the version to be published. SB has made substantial contributions to analysis and interpretation of publications in the field of ICU related diseases, drafting the manuscript, revising it critically for important intellectual content, has given final approval of the version to be published. EC has made substantial contributions to analysis and interpretation of publications in the field of mitochondrial biogenesis in sepsis and possible therapeutic strategies, drafting the manuscript, revising it critically for important intellectual content, has given final approval of the version to be published. PD has made substantial contributions to analysis and interpretation of publications in the field of mitochondrial biogenesis in I/R and possible therapeutic strategies, drafting the manuscript, revising it critically for important intellectual content, has given final approval of the version to be published. LG has made substantial contributions to analysis and interpretation of publications in the field of lipophilic antioxidants and mitochondrial ROS formation, drafting the manuscript, revising it critically for important intellectual content, has given final approval of the version to be published. AVKu has made substantial contributions to analysis and interpretation of publications in the field of reactive oxygen and nitrogen species related to mitochondrial dysfunction, drafting the manuscript, revising it critically for important intellectual content, has given final approval of the version to be published. JT has made substantial contributions to analysis and interpretation of publications in the field of signalling mechanisms related to mitochondrial dysfunction, drafting the manuscript, revising it critically for important intellectual content, has given final approval of the version to be published.
